# Contextualizing the tone of the operating room in practice: drawing on the literature to connect the dots

**DOI:** 10.3389/fpsyg.2023.1167098

**Published:** 2023-06-02

**Authors:** Hillary Lia, Melanie Hammond Mobilio, Frank Rudzicz, Carol-anne Moulton

**Affiliations:** ^1^The Wilson Centre, Toronto General Hospital, University Health Network, Toronto, ON, Canada; ^2^Temerty Faculty of Medicine, Institute of Medical Science, University of Toronto, Toronto, ON, Canada; ^3^Vector Institute for Artificial Intelligence, Toronto, ON, Canada; ^4^Temerty Faculty of Medicine, Institute of Health Policy, Management and Evaluation, University of Toronto, Toronto, ON, Canada; ^5^Department of Computer Science, University of Toronto, Toronto, ON, Canada; ^6^Faculty of Computer Science, Dalhousie University, Halifax, NS, Canada; ^7^Division of General Surgery, University Health Network, Toronto, ON, Canada

**Keywords:** operating room teamwork, interprofessional teams, emotions as social information (EASI) theory, surgical team performance, operating room tone

## Abstract

The study of teamwork in the operating room has made significant strides in uncovering key constructs which shape safe and effective intraoperative care. However, in recent years, there have been calls to understand teamwork in the operating room more fully by embracing the complexity of the intraoperative environment. We propose the construct of tone as a useful lens through which to understand intraoperative teamwork. In this article, we review the literature on culture, shared mental models, and psychological safety, linking each to the construct of tone. By identifying tone as a theoretical orientation to demonstrate the overlap between these concepts, we aim to provide a starting point for new ways to understand intraoperative team dynamics.

## 1. Introduction

The tone of the operating room is an under-studied construct that may shape team dynamics to affect team performance and patient safety. The concept of operating room tone was first identified in a 1986 Perspectives piece in the Canadian Medical Association Journal where Leighton (1986), an anesthetist, described the tone of the operating room as an atmosphere which ranges from tranquil to tense. The nature of tone was argued to be shaped by the degree of civility and collegiality of the interactions between team members, in particular the surgeon and the anesthetist. Leighton (1986) proposed that basic respect and courtesy in the operating room can shape a positive tone and improve teamwork, describing sharing the anesthetic plan with the surgeon as “the first step in setting the tone in the operating room, which is so important for the success of the surgery, not to mention the pleasure of those participating in it” (p. 444). Although a perspective piece rooted in anecdotal experience rather than data, Leighton (1986) work provided the language to identify a construct that was arguably experienced by many but that was—and remains to an extent—unarticulated academically. Since 1986, the tone of the operating room has emerged as a relevant finding in several studies examining factors shaping team dynamics, coordination, and leadership (Leach et al., 2011; Nurok et al., 2011; Stone et al., 2017), indicating that the tone may be an important facet of operating room team dynamics. However, the concept of tone itself has not been the subject of study.

### 1.1. Tone as a peripheral finding

Tone-setting behaviors have been associated with perceived leader effectiveness (Stone et al., 2017), teaching effectiveness (Hauge et al., 2001), and psychological safety (Weller et al., 2018). In a 2017 study, Stone et al. (2017) coded leadership behaviors based on their developed taxonomy and examined the association between the frequency and valence of those behaviors and ratings of perceived leadership effectiveness. Surgeons who enacted positive tone-setting behaviors (i.e., constructive humor, compliments, and reassurance) more often than negative tone-setting behaviors were perceived by other operating room staff members as more effective leaders. Hauge et al. (2001) similarly developed a coding scheme to categorize teaching behaviors. Tone-setting was one of four categories of key teaching behaviors and included both positive and negative behaviors. While this study did not connect to learning outcomes, based on observations, the study suggests that the tone set by the attending surgeon facilitated an environment for learning. Further, in a study of the effective use of the Surgical Safety Checklist, authors reported that senior members in the operating room set the tone for how the checklist is used which can either promote or deter from a sense of psychological safety (Weller et al., 2018). When senior members demonstrated commitment to using the checklist, other team members described increased engagement. Dependent on how the tone is set staff members may be more or less willing to speak up with questions and patient concerns during the administration of the Checklist. The perceived ability, or inability, to speak up during the Checklist may persist throughout the case. The above studies illustrate how leadership behaviors can set the tone for team performance. Since tone is rooted in team interaction, we may, in future, benefit from drawing upon studies of leader communication to inform tone in the operating room. For example, the use of inclusive language by the leader to increase team member voice (Weiss et al., 2018). However, while the above studies described tone-setting behaviors and linked tone to leadership, teaching, and psychological safety, each lacked a clear definition of tone, an explanation of how tone emerges and the mechanism by which it shapes team factors. If we are to understand the effect of tone and begin to draw from the extant literature, we require a deepened understanding of this phenomenon.

While their study did not interrogate the concept of tone specifically, Leach et al. (2011) described tone and its effect in their study of factors associated with professional role affecting teamwork in the operating room. The authors found that the operating room has an enacted environment, the tone, that changes in response to team interactions and events of the operation. The attending surgeon was primarily responsible for setting the tone though other team members, notably the circulating nurse prior to surgeon entry to the operating room, had a role in shaping the tone of the operating room. The study revealed that some teams made a deliberate effort to create a calm tone to maintain control, specifically in response to complication. Dependent on the tone of the operating room, the teams’ ability to adapt and exhibit coordination changed, particularly in response to stressful and unexpected events. Though the tone was not the focus of this study, its identification within the results provides insight into this team construct which we can build upon.

Based on the existing literature, the tone of the operating room may influence how teams dynamically come together and fall apart throughout an operation. However, all studies reviewed lack a clear definition of tone and understanding of how it emerges and shapes teamwork in the naturalistic operating room environment. In this review, we integrate the findings in the literature to define the tone as a dynamic construct which describes the affective atmosphere among team members, rooted in shared understanding of procedural requirements and team norms.

### 1.2. Tone as an emergent state

We conceptualize tone as a team emergent state. Team emergent states (TES) are constructs that describe team properties or characteristics in a dynamic fashion. TES emerge from team context and processes through interactions among team members resulting in phenomena or effects that are greater than the sum of its parts (Cronin et al., 2011; Waller et al., 2016). Fundamental to TES, lower-level interactions and phenomena converge and, in some cases, there is mutual causality, where the lower-level interactions and constructs that the TES emerged from are then influenced by the TES (Waller et al., 2016). Emergent phenomena have four properties: they are *global*, emerging from lower- or micro-level components, *coherent*, enduring over time, *ostensive*, they are experienced and recognized by team members, and of *radical novelty*, as their features cannot be fully reduced into lower-level components (Waller et al., 2016). TES as a framing allows for the interrogation of complex phenomena that are rooted in social interaction.

We propose that tone is a TES, emerging from team interaction, enduring over time with changes based on interaction, highly influenced by context, experienced by team members in the work environment and irreducible into its lower-level counterparts. Thus far, the tone of the operating room has not been studied formally as an emergent state. We propose that this framing will aid researchers in understanding and formalizing the construct of tone by providing a language for the way in which tone can be seen as a complex interaction of context factors and constructs, rooted in team interactions.

Using the lens of TES, we formalize the construct of tone in this review, demonstrating how known team constructs inform the study of tone while they themselves are influenced by the tone. To do so, we use Emotions as Social Information (EASI) theory as lens for understanding the team interactions that lead to tone emergence. Using this framework, we review three team constructs that influence and are influenced by tone: culture, shared mental model, and psychological safety. We use EASI to demonstrate how team constructs can influence how social interactions are perceived and enacted leading to tone emergence. Though there are many constructs that may inform and be informed by tone, the EASI framework allows us to focus our analysis on the mutually causal relationship between tone, culture, shared mental models, and psychological safety.

## 2. Tone and Emotions as Social Information (EASI) theory

Emotions as Social Information (EASI) theory is a framework which describes and predicts how outward emotional expressions are processed and understood to affect behavior at the interpersonal level (Van Kleef, 2009). EASI has been used to understand interpersonal interaction in a variety of contexts (Homan et al., 2016; Wang et al., 2018; van Kleef et al., 2019). The premise of EASI theory is that emotional expressions provide information to the observer which can influence subsequent behaviors, attitudes, and cognitions. The processing of information is moderated by contextual factors that affect affective reactions and inferential processes which ultimately lead to the response of the observer.

We have previously augmented the EASI framework with contextual factors in the operating room to propose a mechanism through which we can understand tone emergence (Lia et al., 2022). Using the framework of EASI we demonstrated how key team constructs (shared mental model, culture, and psychological safety) shape how interpersonal behaviors are processed and understood to affect subsequent behavior. We argued that by studying tone through this theoretical framework, we may begin to more precisely understand what the tone is, how it is emerges and what effect is might have on teams (Lia et al., 2022). In our previous work, we suggested that the study of tone may demonstrate how EASI as an individual-level framework may be extended to understand the group. Here, we will expand our work by exploring in depth the three related constructs that have been identified within the EASI framework as critical to team performance (culture, shared mental models, and psychological safety) and their relationship to tone. While there exist many team constructs which may be related to tone, we select this subset of three constructs to demonstrate how tone is situated in the broader context and how it can be studied in relation to the existing literature.

We use the EASI framework to understand how team interactions are influenced, processed and understood in the operative environment to produce tone. Our understanding is facilitated by the incorporation of known team constructs, culture, shared mental model, and psychological safety. First, we study culture and its’ relationship to tone. According to EASI, team interactions are processed and understood by the observer to produce an output behavior, cognition and/or attitude. Culture shapes the perceived acceptability and meaning of social interaction (Hofstede et al., 2010), thus we propose that culture is essential to understanding team interactions and the manner in which they are processed to shape team interactions, and, ultimately, tone. Next, we examine shared mental model and its relationship to tone. The essence of EASI is that emotional displays convey information about the task or social expectations (McComb and Simpson, 2014), suggesting that the shared understanding of the situation, or shared mental model, is upheld by interpersonal interaction. We study shared mental model with tone to understand how these constructs are inter-related and how they may emerge through interpersonal interaction. Finally, we review psychological safety and how this construct is shaped by and shapes the tone. As described, the EASI framework conveys information about social expectations and team norms. Thus, the degree of psychological safety experienced, or perceived ability to speak up, share ideas and ask questions (Edmondson and Lei, 2014), may be modified by emotional expressions of team members. In this review, we explore how tone may influence and be influenced by the degree of psychological safety experienced.

The purpose of this review is to situate the construct of tone in the literature by outlining the mutual causality between tone and other key team constructs to integrate the literature on teams. We will expand our work by exploring in depth the three related constructs that have been identified within the EASI framework as critical to team performance (culture, shared mental models, and psychological safety) and their relationship to tone to demonstrate how the literature informs tone and how the study of tone contributes to the literature. Finally, we suggest that a fulsome understanding of tone can in fact connect individual team constructs to contribute an integrated understanding of team dynamics in the operating room.

## 3. Culture

Culture captures the shared patterns of thinking, feeling, and acting in societies, organizations, and groups (Hofstede et al., 2010). Culture can be described using six dimensions: power distance, uncertainty avoidance, individualism vs. collectivism, masculinity vs. femininity, long term orientation, and indulgence vs. restraint (Hofstede et al., 2010). In work groups, culture is an important social force that shapes group membership and provides a framework for individuals to understand social interactions and group expectations (Hofstede et al., 2010). In the workplace, culture includes routines and norms that guide appropriate or expected behavior (Hemmelgarn et al., 2006). These six dimensions may be examined within the operating room context to understand how covert values and norms shape team interaction and, ultimately, the tone. We propose that the Hofstede model may be used in future empirical study to characterize the unique operating room culture and provide contextual information that explains the tone.

The current literature on operating room culture describes a unique environment shaped by hierarchy (high power distance), rigid expectations for adherence to standards and guidelines of practice (high levels of uncertainty avoidance), and the high stakes, interdisciplinary nature of surgery. In the operating room, an emphasis on knowledge and competence defines culture and social structures (Gillespie et al., 2008). The surgeon is viewed at the top of hierarchy, followed by anesthetists, then nursing staff and other professions; however, the social order may shift depending on the expertise required in the context of the situation at hand (Gillespie et al., 2008). Moreover, there exist hierarchies within the non-surgical professions where individuals who demonstrate specialized knowledge are rewarded with opportunities to participate in more challenging operations and, over time, build relationships with surgeons (Gillespie et al., 2008). These relationships have the potential to push certain staff members up the hierarchy as their expertise and knowledge extends to predicting surgeon needs in various scenarios. This increased knowledge is further valued as it facilitates smooth and efficient conduct of the operating room. An understanding of how demonstrated knowledge and competence define culture to shape the social order and team interaction may contextualize the interactions which set the tone: how meaning is made in social interaction, whose social behavior exhibits the most influence, and why key members are influential in shaping the tone.

We may use the Hofstede et al. (2010) dimensions of culture to formally integrate the described literature as a means of understanding the norms and values of operating rooms in general, as well as in specific institutions. This deepened understanding of culture in the operating room can provide a lens through which we understand how team members interact and, emerging from their interaction, the tone. Culture and tone similarly underpin the unwritten rules governing social conduct in the operating room, however, they are distinct in their dynamicity. Culture is long-standing and generally resistant to change (Hofstede et al., 2010) while tone changes from moment to moment in the operating room. We propose that as culture governs generally accepted behaviors and ways of being, the culture may shape the range of tones which are set in the operating room and may help us understand how and why tones change, how behaviors change the tone and how the tone changes subsequent behaviors.

While we may better understand the tone using the lens of culture, developing an in-depth understanding of tone may in fact contribute to the literature on culture. The tone, being dynamic and distinct though shaped by culture, may be described as the *enactment* of culture. We may better understand how culture is navigated in real time by understanding social interaction in the operating room using the dynamic lens of tone. While culture is typically measured using cross-sectional surveys or qualitative methods, the lens of tone may provide a tool to better understand those results in the real environment.

## 4. Shared mental model

Shared mental models are knowledge structures among team members that represent a shared understanding of the task, expectations for the task and explanations for events and behaviors related to the task (Cannon-Bowers et al., 1993). Shared mental models allow for coordination among team members as they work toward their goal (Cannon-Bowers et al., 1993). The perceived leader of the team has a critical role in the development of a shared mental model and it has been suggested that training team leaders’ skills for fostering shared mental model could strengthen team performance (Cannon-Bowers et al., 1993).

A concept analysis of shared mental models in healthcare collaboration by McComb and Simpson (2014) describes the defining attributes, antecedents and consequences of shared mental models in healthcare teams. For shared mental model to emerge, two or more individuals must be working on a shared task, there must be communication among these individuals, and the individuals must possess knowledge of the context, including roles of each member, relevant protocols, and task requirements. Shared mental models have four defining attributes: content, similarity, accuracy, and dynamics (McComb and Simpson, 2014). The content of a shared mental model has two domains: the teamwork domain represents an understanding of who the team members are, their capabilities, team expectations and norms while the taskwork domain represents an understanding of the shared goal, progress toward the goal, understanding of next steps and potential for error and complication. The similarity attribute represents the degree to which the mental model aligns among the individuals of the team while the accuracy attribute describes the degree to which the shared mental model reflects the reality of the situation at hand. Finally, the dynamics attribute describes the response of the team to changes in the team environment requiring updates to existing mental models. When an effective shared mental model is in place, it can increase motivation, facilitate task-related processes and improve performance. However, mental models may not align among team members, often related to poor communication, and can undermine team processes and performance (McComb and Simpson, 2014; Wilson, 2019; Gjeraa et al., 2022).

The tone of the operating room may be understood using shared mental model as a construct. For instance, McComb’s framing of shared mental model includes team expectations and team norms under the teamwork domain. A “tense” tone in the operating room may indicate that social chatter is not accepted at that moment whereas it may be invited when other tones are present. Moreover, the taskwork domain of shared mental model includes a shared understanding of progress toward a shared goal. A sudden switch to a more focused tone may indicate that an unexpected obstacle or challenge has occurred and may prompt team members without a view of the operative field to seek information about what has occurred and initiate procedures to support the surgical sub-team. We may better understand and contextualize tone by honing in on the shared mental model to understand how the events of the case shape what tone is set. Moreover, we may examine instances where mental models among team members are in fact mismatched and observe how this phenomenon shapes the tone in the operating room. By drawing upon McComb’s framing of shared mental model, we may begin to understand tone in greater context. The tone may bridge the procedural aspects of the case to the affective and social components of work in teams.

Additionally, the tone of the operating room may contribute to the literature on shared mental model. The dynamic nature of the tone may provide a window with which we can understand how shared mental models are maintained and how teams negotiate deviations in shared mental model. The tone may shift in response to events of the operation and may prompt team members to update their mental model. For instance, if the attending surgeon suddenly engages in external behavior that changes the tone, other staff may infer that there was a change in procedural requirements and begin to seek information to update their shared mental model. By understanding how tone shifts with events of the operation, we may understand how individual team members interact, gather information, and initiate work processes to support collective efforts.

## 5. Psychological safety

Psychological safety is a construct that describes the perceived consequence of an individual taking interpersonal risks in a given context (Edmondson and Lei, 2014). This is typically studied in terms of sharing ideas, speaking up, asking questions, and learning. Psychological safety has been studied in a variety of organizations across industries and has been found to be a key factor for facilitating team coordination and performance (Edmondson and Lei, 2014). In the healthcare setting, the degree of psychological safety experienced at work can affect job performance, job satisfaction and rates of turnover (Grailey et al., 2021).

In the healthcare environment, teams are influenced by pre-existing hierarchies which can prevent the sharing of knowledge and open communication from team members occupying lower positions in the hierarchical structure (Nembhard and Edmondson, 2006). Low psychological safety among these team members can be responsible for medical error, reduce opportunities for team learning, and negatively impact patient safety (Nembhard and Edmondson, 2006). Team leaders have a crucial role in facilitating a sense of psychological safety (Nembhard and Edmondson, 2006; Edmondson and Lei, 2014; Grailey et al., 2021). Leaders who are perceived as more inclusive moderate the relationship between lower status and psychological safety by creating an environment conducive and receptive to speaking up behaviors (Nembhard and Edmondson, 2006). Grailey et al. (2021) suggest that leaders in the healthcare context can learn to understand situations which lead to sense of low psychological safety and modify their own behavior to facilitate speaking up behaviors and invite open discussion.

We may understand tone emergence and changes in the operating room by examining psychological safety in the operating room. We may understand why particular tones arise in certain environments and not in others based on the psychological safety experienced in those environments. We may additionally better understand how team members interact, navigating power structures in their work environment, to affect the tone. By incorporating psychological safety into the study of tone, we may better understand how and why some voices are heard and others are not.

As with culture and shared mental model, the dynamic nature of tone can inform the study of psychological safety. While psychological safety is typically studied as a dynamic construct using cross-sectional surveys, Amy Edmondson, who coined the term, calls for the study of psychological safety as a dynamic construct, emphasizing that the construct evolves over time (Edmondson and Lei, 2014). The tone of the operating room changes over time, perhaps in response to the psychological safety and perhaps affecting psychological safety. We propose that the study of tone may answer calls to understand the dynamic nature of psychological safety using tone as a lens.

## 6. Discussion

In this review, we demonstrate how key team factors can be used to understand tone: what it is, how it may be interpreted, and how it influences team performance. Though the tone of the operating room has been discussed in the literature, in-depth exploration of tone itself has not been the focus of research to date. A rich understanding of intraoperative tone as a theoretical construct has the potential to scaffold our understanding of OR culture. By better understanding the moment-to-moment tone of the OR, we may gain greater appreciation for how the underlying construct shapes and is shaped by teamwork in response to unexpected challenges, changes in staffing, and nuances in the relationships between staff members.

We describe how the shared mental model of the operating room may shape and be shaped by tone. Unlike culture, which is persistent and pervasive, tone can change depending on factors such as the phase of the case and situational characteristics. These “moments” of change in tone may afford the opportunity for researchers to glimpse the impact of culture on teamwork in new ways. For example, while tone may shift during a case, the particular ways in which the shift occurs is likely to remain within the boundaries of what is culturally appropriate. A deepened understanding of tone might allow researchers the opportunity to access the intersection of these moments of cultural influence on OR interactions through the use of qualitative methodologies. Better understanding the emergence and impact of tone may also help to provide an orientation for researchers interested in OR culture to ask questions related to Lingard (2016) collective competence, and the limits of individual expertise on teamwork in the operating room.

Similarly, understanding psychological safety as a dynamic construct shaped by tone can contextualize team interaction and outcomes to help researchers better understand this construct and how it can be modified to improve patient safety. For example, although the surgeon has been identified as the leader in the operating room (Gillespie et al., 2008), their voice is rarely heard in discussions around psychological safety. While it is critical to recognize the impact of hierarchy in psychological safety, the cascading impacts of preexisting OR hierarchies (Gillespie et al., 2008) are rarely appreciated in the context of the responsibilities of the surgeon in practice. Tone may help us understand how surgeons, as leaders, actively balance the expectation to provide an environment conducive to psychological safety for team members alongside other responsibilities, such as patient safety and institutional expectations. Given that the tone of the operating room may convey information about who can speak up, when they speak up and what they speak up about, tone may provide a lens with which to understand how psychological safety evolves dynamically in team interaction. The study of tone may respond to calls in the literature to move from the study of psychological safety as a static construct to the study of psychological safety as a dynamic construct to capture nuances such as shifts in time (Edmondson and Lei, 2014).

Team constructs can be integrated with the EASI framework, as demonstrated in our earlier work (Lia et al., 2022), to provide theoretical insight into how interpersonal interactions are understood and shape team behaviors. An augmented EASI framework can provide insight into how individual team constructs interact in the real work environment to shape teamwork. This builds upon the existing literature by providing a means to study teams in a manner that examines the team as whole rather than positioning the team as the sum of its parts. This approach to the study of team constructs additionally contributes to our understanding of team constructs that were previously difficult to capture and conceptualize as dynamic constructs. By studying tone, we draw from key team constructs, inform each construct and connect them to develop an integrated understanding of team performance. We argue that the study of tone can draw together team constructs in the operating room, bringing them from individual facets of understanding toward a holistic interpretation of team performance in the operating room. Tone may be the mortar describing how the “bricks in the wall” of teamwork (i.e., known team constructs) are connected. By connecting these bodies of literature, we propose that tone may be understood and studied in a manner which emphasizes and appreciates the complexity of the operating room environment. The study of tone in this manner can elucidate the qualities and characteristics of high-quality teamwork so that we may train operating room staff members to maximize productive team interaction and minimize those that deter from safe and efficient intraoperative care.

We propose that to provide this robust understanding of tone, a mixed methods approach is essential. As a first step, a detailed theoretical understanding of tone needs to be developed to define the tone. This framework may be augmented with an exploration of how tone is experienced by staff members in the naturalistic environment to provide greater insight. We may move from theoretical to practical by using a theoretical basis for tone to understand team interactions *in vivo* to study how tone emerges, changes, and is sustained in the workplace. This study may quantitatively explore team interaction and changes in tone, perhaps using the lens of affect. By understanding tone as a phenomenon both theoretically and practically, we might uncover how the collective comes together and falls apart at critical moments (Lingard, 2016). This knowledge may, in future, allow for new facets for team skills training to ensure heightened and sustained safety and efficiency in the operating room; as well as provide important insight into the literature around psychological safety and team affect.

## Author contributions

All authors listed have made a substantial, direct, and intellectual contribution to the work, and approved it for publication.
